# Concept, Content, and Context Perspectives of Quality of Agrofood Products: Reflections on Some Consumer Decision-Making-Purchase Scenarios

**DOI:** 10.3389/fnut.2020.578941

**Published:** 2020-11-13

**Authors:** Charles Odilichukwu R. Okpala, Małgorzata Korzeniowska

**Affiliations:** Department of Functional Food Products Development, Faculty of Biotechnology and Food Science, Wrocław University of Environmental and Life Sciences, Wrocław, Poland

**Keywords:** agrofood product, agrofood industry, interaction, information, decision-making, consumer quality

## Abstract

Quality attributes in agrofood products can be somewhat difficult to identify and observe. The quality of the same agrofood product in two different market shelves would, most likely not be exactly the same when compared to each other, even if both belong to the same batch. There are quality attributes peculiar/specific to one product, which stands it unique from the other. The basics/fundamentals underlying such peculiarities/specificities can be found either in concept, content, and context perspectives of quality. It appears however that no publication has deliberated on these three aspects together, that is, concept, content, and context perspectives of quality of agrofood products, particularly on how it contributes to the decision-making to purchase an agrofood product. We, therefore, in this current work, looked at concept, content, and context perspectives of quality of agrofood products, specifically discussing some reflections on some consumer decision-making purchase scenarios. Each of these, “concept,” “content,” and “context” perspectives independently project very important meanings to the quality of agrofood products. There appears a thin line that would separate concept, content, and context perspectives of quality in the choice/decision-making of purchase of agrofood products. To solely depend on either concept, content, or context perspective of quality will likely provide the consumer with insufficient information about the (given/specific) agrofood product. Interaction between any two will most likely improve the information. Obviously, the interaction between the three, would most likely provide sufficient information about the quality and help consumers make a more informed decision of purchase.

## Introducing Quality in Agrofood Products

Quality should neither be perceived as a physical entity or instance with a fixed position in space and time, nor a scientific or technical word (1–3). Quality, the very useful idea in general life and management (3), represents a set of characteristics of a product (or service) that provides some ability/potential to meet up with consumers' expressed/implied needs (4). However, quality attributes in agrofood products can be somewhat difficult to identify and observe (5). To either envisage or picture the quality particularly in terms of the composition of properties, however, the differences in quality can potentially depict either: (1) changes in the markedness of one or more properties in quantity (vertical/product modification of quality), (2) decreases/extensions in the values (horizontal product modification), or (3) changes in how value associates with single components (consumer induced quality change) (6). Whilst the estimates of quality of a given agrofood product can be drawn up either objectively or subjectively (7), such (estimates) can equally vary either from location-to-location and or by product-to-product, owing to the complex nature of the (agrofood) global supply chain (8).

## Quality: From Agrofood Product to Industry—A Few Highlights

Quality is an essential element of any existing economic activity, with a direct impact on consumer, producer, and product/service (9). Information asymmetry between buyers and sellers of agrofood products can complicate the buyer's ability to identify with quality and assert guarantees in an institutional form, especially in such situation(s) where there is a need to counteract the effects of quality identification, as well as uncertainty. Besides, a contractual definition of quality would focus on the transition between the buyer and the seller of an agrofood product (5). Nonetheless, quality—an objective continually sought for within the agrofood industry—can be seen in three distinct perspectives, namely: (1) consumer perspective—understanding quality based on experience over time via dimensions of risk and trust; (2) institutional perspective—the use of objective/regulated indicators to define quality largely based on hygiene requirements; and (3) producers' perspective—where both raw materials and production methods help to define the quality of agrofood products (10–12). Moreover, quality can plausibly help in opening up discussions about agrofood products among key supply chain stakeholders, which in the broader sense would be contributing to food standard authorities/boards in building regulatory frameworks. Considering its fluid-/socially constructed nature, quality from the content and context standpoints appears to be with increasing emphasis across the globe. Quality, as an essential strategy for the future development of the farming/food industry, has similarly been echoed by the various agrofood product actors/stakeholders (13).

## Some Consumer-Related Agrofood Product Quality Challenges, and Problems

Meeting the prerequisite product quality benchmark/standard always remains among the key challenges for the agrofood product industry. Indeed, the quality of agrofood products is differentiated by a wide array of factors. The diverse differences in shelf-life time, cost, seasonality, level of processing required to make the product either fit for consumption or increase consumer appeal, degree of freshness especially for those ready after harvest, etc., are among the many factors that individually and or collectively challenge the resultant quality of agrofood products. Consumers have no alternative but to grapple with these diverse factors that affect quality, which are largely done through physical observations prior to making the appropriate decision on whether to purchase the agrofood product(s) or not. According to Dequiedt (14), how consumers see quality attributes of an agrofood product largely rely on three major facets, namely: (1) the experiences acquired after consumption, (2) how consumers search for it (the agrofood product), and (3) the credence associated with it (the agrofood product), which may not always be discovered either before or after its purchase.

Widely understood, the quality of the same agrofood product would likely not be exactly the same in one market shelf compared to the other. Such (quality) differences in agrofood products by markets (shelves) might likely underscore the problems that emanate from how quality is produced, revealed, and certified (14). For example, how quality produced could pose problems is when the same agrofood product from one producer meets the expectations/requirements of one set of consumers but not so for the other because of differences in quality, potentially attributable to variants in emphasis on quality. In addition, how quality revealed could pose problems is if the same agrofood product in two different market shelves differ by price because of differences in quality, wherein the higher quality is pricier than the lesser quality (15). In addition, how quality is certified could pose problems if the third-party mechanisms that facilitate truthful elevation of product (quality) information, allows a given (set of) agrofood product(s) that is clearly of a substandard quality to enter into the market (14). The previously mentioned quality challenges, potentially, could be compromising the consumers' integrity and trust of the agrofood product supply chain. And if such were to worsen, it might likely cumulate into complicated/complex short- and long-term conflicts/problems within the agrofood product supply chain. One can only imagine a conflict in the quality price of agrofood products. In addition, one can only imagine substandard quality agrofood products finding its way into the market shelves. Notably, a substandard agrofood product can potentially pose some health risk to consumers, especially after the “best before” dates (16, 17).

## Justification of this Perspective Paper

Mindful of the previously mentioned consumer-quality related challenges, when a consumer in a supermarket, for example, is about to purchase a given agrofood product, whether it is a bunch of bananas, some fresh tomatoes, fresh fish/meat, tinned fish, or even a loaf of bread, “quality” and its related aspects would most likely be considered. In each given/selected (agro)food product, there are associated quality attributes peculiar/specific to one product, which stands it unique from the other. Also, how Person A will perceive the quality of the same agrofood product will likely not be the same as Person B. The basics/fundamentals underlying such peculiarities/specificities, we believe, can be found either in concept, content, and context perspectives of quality. Therefore, how do these three ideas/perspectives of quality function/operate when a consumer is about to purchase an agrofood product? Do these three ideas/perspectives function/operate independently or interactively? How do these three ideas/perspectives independently or interactively drive consumers in their decision-making to purchase an agrofood product? It appears, however, that no publication has deliberated on these three aspects together, that is, concept, content, and context perspectives of quality of agrofood products, particularly on how it contributes to the decision-making of purchase an agrofood product. We, therefore, in this current work, looked at concept, content, and context perspectives of quality of agrofood products, specifically discussing some reflections on some consumer decision-making purchase scenarios. Subsequent sections will be structured in two parts, namely: (1) concept, content, and context perspectives of quality of agrofood products; and (2) reflections on some consumer decision-making-purchase scenarios, which can be commonly found.

## Concept, Content, and Context Perspectives of Agrofood Product Quality

### Concept Perspective of Quality

“Concept” can be defined simply as a principle or idea, an idea for a new product, and about a particular subject (18). Therefore, the concept of quality can be seen as an idea concerning the function/value ascribed to the character/property of, as in this case, the agrofood product. It can also involve, not only the origin of the product but also how hygienic and safe the food product is (6). It can also provide an avenue to interpret ideas surrounding quality, very applicable to any given agrofood product. As a benchmark to either recognize or separate an agrofood product based on predetermined specification(s), the concept perspective of quality can be either established or identified at any stage of the production/supply chain. In this case, a high level of precaution would have to be applied so as to ensure the consistency of quality control either along with or on each production line/stage (6).

Adapting from de Heer et al. (19) and Mogezomp et al. (20), such terms like “improved,” “optimized,” and “perfect” can be ascribed to the “concept” of quality, which can be applied to agrofood products. If that is to be the situation, “improved” can be when the product has received some added value over a premium one, “optimized” can be based on achieving an enhancement peak on either one or more of the specific product properties, and “perfect” can be when product characteristics attained peak consistently with 100% market response over a substantial time period. The concept perspective of quality can, therefore, be quantified when it gets allocated with a numerical value, which can help generate a “quality” type of data. It is on this basis that the concept perspective of quality plays a useful role in the food industry, to either create or determine the level of consumer acceptability on a given agrofood product (6). Besides being among the most widely studied issues in agriculture, the concept perspective of quality might closely associate with hygiene/health and natural condition of agrofood products (10, 12). Based on the existing food production structures, the creation of new concepts can therefore take place. This would allow the quality of selected/specific (agrofood) products to undergo a (healthy) market competition with another competing similar one, in order to sustain the eventual/overall image of “quality” (of these products) (6).

A consumer looking at an agrofood product in a supermarket shelf for example should not see the concept perspective of quality as either abstract or immaterial. This is because previous studies of Pringent-Simonin and Hérault-Fournier (21) and Becut (22) posited the concept perspective of quality as built up either by economic actors via voluntary agreements (product specification), or through public policy decisions (e.g., minimum quality standards). Becut (22) equally understood “quality” (applicable to agrofood products) could help to increase the competitiveness between items within a given market space. From another perspective also, the concept of quality can be either developed or initiated, based on cultural (signs and symbols associated with specific values associated with a specific items/products), legal (precise norms for intellectual property rights), and political (institutions that manage the certification, protection, and registration systems) platforms (22). In some of its clusters of interpretation, the concept of quality can be interchanged with “local” or “producer” who can be seen as responsible to impart some virtues to the given (agrofood) product. This will help consumers to socially perceive the concept perspective of quality, such that in this way, values like “authentic,” “healthy,” and “traditional” could then be associated with the given (agrofood) product (23).

### Content Perspective of Quality

“Content” can be defined simply as everything that is contained in something (14). In line with this, the content perspective of quality would, therefore, consider the entirety of the information that can be deduced about the given agrofood product. Basically, agrofood products constitute nutrients. In fact, information about its quality is underpinned within its nutritional content. Considering the work of van der Spiegel (24), the content perspective of quality should corroborate well with the physical aspects of (agrofood) product quality, which can be measured, and demonstrated by composition, for example, the content of water. The content perspective of quality can therefore equally serve as a useful candidate to validate the consistency of performance variables of a given agrofood product.

Basically, carbohydrates, proteins, fats and oils, vitamins, minerals, and water constitute the nutritional composition of agrofood products. Considering the content of quality has been associated with the nutritional composition of agrofood products, consumers continue to depend on it taking into account all available/relevant information (25). Through the nutritional constituents, the content perspective of quality plays an important role, especially in the (nutritional) profile of a given agrofood product, which can be classified by food categories/subcategories, the latter largely depends on the profile's (nutritional) ratio (26). Nutritional profiles, in general, would likely have played a key role in developing, for example, the Healthy Eating Index, which measures the diet quality used to assess how well a set of foods associated with key recommendations of Dietary Guidelines for Americans (27, 28).

Another aspect to consider is when consumers look at any given packaged product on a supermarket shelf. Not only would they look at the nutritional contents, but they also look at the labels. Indeed, the content perspective of quality would equally be contributing to developing/establishing (shelf) labels, which allows for dates to get marked, especially in packaged agrofood products. For instance, the expiry dates explain the minimum durability date and can appear in two ways: (1) use up to/use by, or (2) better to use before/best before. Notably, some agrofood products can be exempt from expiry date markings, for example, bakery products (consumed usually 24 h post-production), beverages, chewing gum, cooking salt, synthetic vinegar, sugar, and wines (29).

### Context Perspective of Quality

“Context” can be defined simply as the influences as well as events that explain or can be related to a particular situation (18). Looking at a given agrofood product, context perspectives can, therefore, refer to how one or more events/situations can influence the (product's) overall quality. In his well-cited text about planning for quality, Juran (30) argued that the defect-free characteristics exhibited in a given product can help avoid consumer dissatisfaction. Considering this, the “context” perspectives of quality might be instrumental in creating an understanding that connects the (agrofood) product performance with the degree of consumer satisfaction. Moreover, from the context standpoint, Fellows et al. (31) understood “quality” to depict the meeting up with a laid-down set of criteria, expectations, and specifications either agreed upon or well-established by the consumer toward a given (agrofood) product. In addition, the context of quality can, therefore, provide both consumers and sellers some form of choice to help them determine how quality is either embraced, interpreted, perceived, and or seen.

Previous workers such as van Rijswijk and Frewer (32) and Pinna et al. (33) associated quality, with such terms as “good product,” “natural/organic,” and “freshness,” equally applicable to a given agrofood product. This would suggest that the context of quality has the capacity to help create some kind of descriptors for a given agrofood product. Moreover, Ilbery and Kneafsey (34) specifically linked the quality of the product (and services) to specific regions. Possibly, the context perspective of quality can help identify with the location(s) from which an agrofood product has either emerged from or been prepared. Consider a broad market scenario, it can be that the context perspective of quality may allow for product differentiation to take place in response to its demand (35, 36), which is equally applicable to agrofood products. If the context perspective of quality is to be looked at in a broader scope for agrofood products, it might actually help in understanding how and why several regulatory frameworks/standards develop and thrive across geographical continents/regions. For instance, the European Commission has adopted several regulations on the application of EU quality schemes. Take, for example, the legislation that explains the use of logos, how it is related to each quality scheme, and how such schemes should be approved, which covers the guidelines labels for agrofood products (37). In addition, labeling helps, not only in elevating the quality but also helps in enforcing it (38, 39).

## Reflections on Some Consumer Decision-Making-Purchase Scenarios

Consumers, in the selection of a given agrofood product, are confronted with decision-making processes made in varying time periods, particularly with respect to quality. The set of characteristics/properties that makes up a given agrofood product, in our opinion, can be seen as the foundation, which contributes to define as well as establish the quality of any given agrofood product, even at the time of purchase. Our opinion would agree with Manole et al. (4) who reiterated that quality has the capacity to represent a set of characteristics of, for example, a given agrofood product, which has the overall aim to meet up with consumers' needs. In addition, consumer, institution, and producer scenarios according to Ilbery and Kneafsey (11) can contribute to better the understanding of the quality of a given agrofood product. Therefore, to discuss these three facets of concept, content, and context perspectives of quality (of agrofood product) therefore comes very timely, considering the key role quality plays to the overall food supply chain/market, worldwide (40, 41). In addition, to implement the concept, content, and context perspectives of quality requires consumers to ascribe a certain degree of trust to the given agrofood product. Trust can be generalized (developed without intention), systematic (formalized in the laws/rules, based on institutional power), process based (repeated interactions between individuals/organizations), and personality-based (personal characterization of the individual) (42).

Most, if not all consumers, in making a decision during the purchase of a given agrofood product, are likely to consider at least two if not all of either concept, content, and or context perspectives of quality. Some consumers, depending on their (food quality) knowledge level, may likely possess some awareness, for example, nutritional specifics associated with the given agrofood product. Indeed, consumers might acquire some knowledge through their food-related experiences/exposures (14), which would guide/help them through the decision-making process, either at the point or period of purchase. Similarly, consumers considering buying either fresh fruits and or vegetables in a food retail store, for example, could apply their personal instincts to help them choose/differentiate between one product over the other, and at the same time, considering their perceived concept, content, and context perspectives of quality. For example, customers at the fishmongers/meat butcher's shop(s), in this instance, could make the effective use of their personal instincts to perceive/view concept, content, and context perspectives of quality.

A consumer preference if consistent to a specific agrofood product over another competing one shows a strong agreement with the (specific product) performance. This could be a reflection on how that specific consumer might have embraced, interpreted, and perceived (31) the quality of that specific agrofood product. Many consumers, in reality, are likely to take some time to check the (agro)food package labels, product (nutritional) content as well as date markings in the process of purchase. Some scenarios may well take place where a consumer who does not usually check these, is being accompanied by another who possess better/increased knowledge about that given specific agrofood product. Sharing knowledge is able to strengthen the other's decision-making. Whether the decision-making to purchase a given agrofood product is self, or influenced by another, there would always be some interaction between concept, content, or context perspectives of quality. Another instance is that perspectives of quality could have some influence based on where the agrofood product is made/prepared and the success of its sales. An example that can fit well is pizza. It is now so diverse and found in many parts of Europe. Pizza makers largely adjust and modify the emergent/resultant product quality to suit the traditional choices of the target locality/population so as to achieve optimum sales. The same pizza from one maker will likely differ from another on the same street! Consumers at the purchase of pizza effectively interact with the concept, content, and context perspectives of quality. Most likely, consumers' experience after consumption will either persuade or dissuade their return to the same pizza shop. In order to meet consumer expectations (31), pizza makers have to establish their product quality (as well as service to that specific region) (34), which increases the product competitiveness at the market place (22).

**Figure 1:**
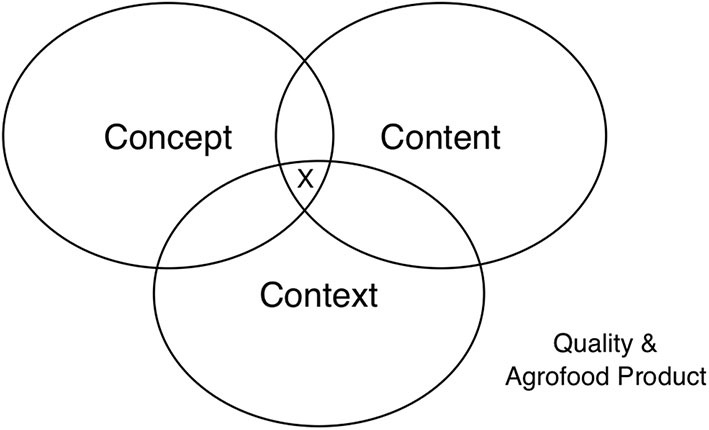
A diagrammatic representation of interaction space between concept, content, and context perspectives of quality of a given agrofood product. The interaction between the three, indicated with “X,” that is, concept vs. content vs. context perspectives of quality.

A diagrammatic representation of interaction space between concept, content, and context perspectives of quality of agrofood product is shown in Figure 1. There appears a thin line that would separate concept, content, and context perspectives of quality in the choice/decision-making of agrofood products. Specifically, solely depending on either concept, content, or context perspective will provide the consumer with insufficient and limited information about the quality of the given agrofood product, in order to make the appropriate decision on whether or not to purchase. An interaction between any two, which could be either, concept vs. content, concept vs. context, or content vs. context perspectives will most likely improve information about the quality of the given agrofood product. Obviously, the interaction between the three, indicated with “X” in Figure 1, that is, concept, content, and context perspectives of quality, would most likely provide sufficient information about the quality and help consumer make a more informed decision of purchase. As a result, the consumer's participation in the decision-making process of purchase will be strengthened, which would help achieve the desired as well as preferred choice of an agrofood product, to help meet up with (specific) demands/needs. In addition, consumers may not realize when concept and content, concept and context, and or content and context perspectives of quality might have actually interacted, particularly in their decision-making process to purchase a given agrofood product.

## Concluding Remarks

Concept, content, and content perspectives of quality are very relevant to any given agrofood product. There appears a thin line that would separate concept (principle or idea about something), content (everything contained in something), and context (influence and events related to a situation) perspectives of quality in the choice/decision-making of agrofood products. In the view to enhance the choice and decision-making of a given agrofood product, there should always be some interaction between concept, content, and context perspectives. Considering that decision-making is one of the factors influencing consumer preference to quality, other factors for future works need considerations, including price, economic status of buyers, season, media, advertisement, availability, etc. Future works reflecting on how these other factors connect with consumers' concept, content, and context perspectives of quality of agrofood products are required, which can help in delineating more pieces of information influencing the choice/decision-making processes of quality of agrofood products, so as to make them more appropriate at the time of purchase.

## Data Availability Statement

The original contributions presented in the study are included in the article, further inquiries can be directed to the corresponding author/s.

## Author Contributions

The idea was conceived and developed by CORO, who prepared the initial draft. MK corrected and edited the article. All the involved authors approved the final manuscript.

## Conflict of Interest

The authors declare that the research was conducted in the absence of any commercial or financial relationships that could be construed as a potential conflict of interest.
